# The predictive value of hemogram parameters for early preterm delivery in pregnant women undergoing cervical cerclage

**DOI:** 10.1590/1806-9282.20240030

**Published:** 2024-08-16

**Authors:** Ümran Kılınçdemir Turgut, Ebru Erdemoğlu, Cem Dağdelen, Mehmet Okan Özkaya, Mekin Sezik

**Affiliations:** 1 University of Health Sciences, Adana City Training and Research Center, Department of Obstetrics and Gynaecology-Perinatology - Adana, Turkey.; 2 Suleyman Demirel University, Faculty of Medicine, Department of Obstetrics and Gynecology - Isparta, Turkey.

**Keywords:** Preterm birth, Cervical cerclage, Neutrophil lymphocyte ratio, Inflammatory markers, Perinatology

## Abstract

**OBJECTIVE:**

This study aims to investigate the predictive value of hemogram parameters in early preterm delivery (32 gestational weeks and below) among pregnant women who have undergone cervical cerclage, based on cervical changes determined before the cerclage procedure.

**METHODS:**

Between 2010 and 2020, a total of 161 patients underwent cervical cerclage. The participants were divided into three groups. Group 1 (n=92) consisted of pregnant women who underwent prophylactic cerclage. Group 2 (n=31) included those with cervical shortening (<5 mm) and/or dilation (≤3 cm). Group 3 (n=38) comprised pregnant women with cervical dilation >3 cm. Each group was further divided based on delivery weeks, with a cutoff at 32 weeks. Demographic parameters and laboratory parameters were assessed.

**RESULTS:**

In Group 1, all hemogram parameters showed no significant differences between deliveries below and above 32 weeks. In Group 2, the neutrophil-to-lymphocyte ratio value before cerclage was higher in the early preterm delivery group (p=0.002), with a cutoff value of 4.75 in receiver operating characteristic analysis. In Group 3, the white blood cell value before cerclage was higher in the early preterm delivery group (p=0.005), with a cutoff value of 13.05×10^3^/μL in receiver operating characteristic analysis.

**CONCLUSION:**

The use of hemogram parameters to predict early preterm delivery in pregnant women undergoing prophylactic cerclage is not appropriate. However, neutrophil-to-lymphocyte ratio value can predict early preterm delivery when cervical dilation is 3 cm or less and/or cervical shortening is 5 mm or less. When cervical dilation exceeds 3 cm, the white blood cell value is more appropriate for predicting early preterm delivery.

## INTRODUCTION

Cervical insufficiency is a rare condition, impacting around 1% of pregnancies and frequently leading to premature delivery^1^. Cervical cerclage procedures are employed to extend pregnancy; however, accurately predicting the success of this surgical intervention can be challenging. The examination of inflammatory markers in maternal blood and/or amniotic fluid is currently underway to assess their potential in predicting the progression of pregnancy. Indeed, there are publications indicating that intra-amniotic infections can contribute to premature delivery^2,3^. Certainly, some previous studies have proposed the significance of assessing infection markers in amniotic fluid through amniocentesis before the placement of emergency cervical cerclage^4,5^. Inflammatory markers such as C-reactive protein, white blood cell (WBC) count, and neutrophil-to-lymphocyte ratio (NLR) have been the subject of extensive research for their role in predicting pregnancy outcomes in women undergoing cerclage procedures^6^.

White blood cell count has long been employed as an inflammation parameter. NLR has garnered attention in recent years as an inflammatory marker with potential predictive value in various medical conditions. It is a straightforward parameter for assessing the inflammatory status and has been utilized as a prognostic marker in cardiovascular diseases^7^, determining the prognosis in some cancer types^8^, as well as in cases of infection and inflammation^9^. The NLR ratio is defined as 1-3 normal, 6-9 mild stress, and above 9 moderate and severe stress^10^, and the clinical significance for NLR between 3 and 6 is uncertain.

The objective of this study is to assess the effectiveness of hemogram parameters in predicting early preterm delivery in pregnant women undergoing cervical cerclage.

## METHODS

### Participants and study design

This retrospective clinical study took place at a tertiary center from September 2020 to December 2020, receiving approval from the local ethics committee (document number 71556). The study included patients undergoing cerclage surgery between 2010 and 2020, adhering to specific criteria.

Inclusion criteria comprised having a singleton pregnancy, undergoing cerclage using the McDonald procedure, delivering at the same hospital, and delivering due to the onset of labor. Exclusion criteria included multiple pregnancies, known uterine anomalies, delivery within 48 h after the cerclage procedure, fetal anomalies, other systemic infections, conditions leading to elevated inflammatory parameters in the mother, and signs of chorioamnionitis.

A total of 201 pregnant women were screened, with 161 meeting the criteria and enrolling in the study. Participants were divided into three groups based on cervical length and/or the degree of cervical dilation.

Group 1: Comprising 92 pregnant women receiving prophylactic cerclage based on obstetric history, without evidence of cervical shortening or dilation.

Group 2: Consisting of 31 pregnant women who underwent cerclage due to cervical shortening (≤5 mm) and/or dilation (≤3 cm).

Group 3: Including 38 pregnant women who underwent cerclage due to cervical dilation exceeding 3 cm.

The groups were further analyzed by dividing them into two subgroups based on weeks of delivery, with a cutoff value of 224 days (32 weeks). Hemogram results were compared between individuals delivering before or after the 30-s gestational week.

### Surgical and medical procedures

Tocolytic treatment was administered to every patient scheduled for the cervical cerclage procedure, starting just before the procedure and continuing for 48 h post-procedure. Tocolytic therapy included either indomethacin (100 mg rectally followed by 25 mg orally four times a day) or nifedipine (initiated at 30 mg/h followed by 10 mg orally every 4 h). The cerclage procedure used the McDonald technique, employing Mersilene suture material. Following cerclage application, each pregnant woman received progesterone gel at a dose of 90 mg administered vaginally once daily until delivery.

In cases where amniotic sludge was detected along with cervical changes, broad-spectrum antibiotic treatment was administered to selected patients in our clinic in mid-2021. Pregnant women included in the study did not receive broad-spectrum multi-antibiotic therapy.

### Demographic parameters and evaluated variables

Demographic parameters included maternal age, gravidity, and parity. Evaluated clinical parameters included gestational age at cerclage placement, gestational age at delivery, days of hospitalization post-cerclage application, and duration of pregnancy prolongation from cerclage application (latency period). Evaluated laboratory parameters included WBC, neutrophil, lymphocyte, NLR, and hemoglobin values just before cerclage application, 6 h after cerclage application, just before delivery, and 6 h after delivery.

### Statistical analysis

Data were analyzed using IBM SPSS version 23. Prior to statistical analyses, checks were conducted to ensure no data entry errors and to assess parameter adherence to expected ranges. Normality assumptions of continuous variables were examined using the Kolmogorov-Smirnov test, and the homogeneity of variances was assessed using Levene's test. Descriptive statistics, including mean and standard deviation, were provided for continuous variables. In cases of non-normal distribution, the Mann-Whitney U test was employed. Receiver operating characteristic (ROC) analysis was applied to significant findings. A significance level of p<0.05 was considered for all analyses.

## RESULTS

Demographic and evaluated parameters are presented in Tables 1, 2, and 3. Across all groups, except for the cerclage application-latency period, maternal age, gravid, parity, hospitalization (days), and gestational days at cerclage application were statistically similar between those delivering earlier and later than 32 weeks. The cerclage application-latency period was significantly shorter in deliveries below 32 weeks of gestation (p≤0.001).

**Table 1:** Comparison of hemogram and demographic parameters according to the gestational weeks of delivery in group 1.

Group 1		Gestational weeks of delivery		p
		≤224 days (n=12)	>224 days (n=80)	
Mean±SD				
	Maternal age (years)	37.42±4.21	35.26±6.34	0.243
	Gravid	4.25±2.66	2.67±1.23	0.065
	Parity	0.88±0.84	0.98±0.71	0.679
	Hospitalization (days)	1.75±0.62	1.80±0.97	0.768
	Gestational days at cerclage applied	99.25±9.21	97.43±7.97	0.572
	Cerclage application-latency period (days)	86.17±40.05	166.44±13.76	<0.001*
Before cerclage application				
	WBC×10^3^/μL	9.87±3.22	9.34±2.13	0.459
	Neu×10^3^/μL	7.80±2.91	6.88±1.90	0.144
	Lym×10^3^/μL	1.82±0.64	1.94±0.52	0.323
	NLR	4.68±2.51	3.79±1.97	0.164
	Hbg g/dL	12.04±1.01	12.43±1.03	0.217
Six hours after cerclage application				
	WBC×10^3^/μL	11.77±3.47	9.95±2.66	0.112
	Neu×10^3^/μL	9.58±3.69	7.87±2.60	0.202
	Lym×10^3^/μL	1.77±0.63	1.64±0.53	0.645
	NLR	6.34±4.22	5.35±2.77	0.755
	Hbg g/dL	11.22±1.16	11.42±1.16	0.501
Before delivery				
	WBC×10^3^/μL	12.93±5.40	10.26±2.71	0.100
	Neu×10^3^/μL	10.39±5.61	7.68±2.34	0.173
	Lym×10^3^/μL	1.83±0.72	1.95±0.50	0.352
	NLR	6.66±4.67	4.07±1.29	0.274
	Hbg g/dL	11.60±1.27	12.26±1.47	0.111
Six hours after delivery				
	WBC×10^3^/μL	16.02±3.98	14.82±4.31	0.415
	Neu×10^3^/μL	14.15±3.90	4.13±12.5	0.207
	Lym×10^3^/μL	1.39±0.61	1.46±0.49	0.563
	NLR	12.18±7.07	10.08±6.64	0.320
	Hbg g/dL	10.16±0.82	11.33±1.72	0.013*

WBC: white blood cell; Neu: neutrophil; Lym: lymphocyte; NLR: neutrophil/lymphocyte ratio; Hbg: hemoglobin.

* p<0.05.

**Table 2:** Comparison of hemogram and demographic parameters according to the gestational weeks of delivery in group 2.

Group 2		Gestational weeks of delivery		p
		≤224 days (n=11)	>224 days (n=20)	
Mean ± SD				
	Maternal age (years)	33.64±8.08	30.55±6.13	0.338
	Gravid	1.88±0.84	2.50±1.59	0.569
	Parity	0.75±0.88	0.81±0.91	0.928
	Hospitalization (days)	3.91±3.18	2.15±0.93	0.072
	Gestational days at cerclage applied	137.27±34.57	137.85±30.15	0.974
	Cerclage application-latency period (days)	32.45±21.21	125.85±35.65	<0.001*
Before cerclage application				
	WBC×10^3^/μL	13.19±5.82	10.58±2.52	0.157
	Neu×10^3^/μL	10.41±6.34	8.06±2.16	0.104
	Lym×10^3^/μL	2.19±1.99	1.96±0.45	0.197
	NLR	7.99±5.40	4.24±1.43	0.002*
	Hbg g/dL	11.23±0.79	12.27±1.28	0.018*
Six hours after cerclage application				
	WBC×10^3^/μL	14.83±5.77	10.77±2.15	0.042*
	Neu×10^3^/μL	12.44±6.05	8.32±2.28	0.048*
	Lym×10^3^/μL	1.52±0.58	1.84±0.52	0.188
	NLR	10.12±8.17	4.98±2.45	0.048*
	Hbg g/dL	10.43±1.17	11.04±1.47	0.410
Before delivery				
	WBC×10^3^/μL	13.37±3.34	10.51±2.05	0.072
	Neu×10^3^/μL	10.83±3.19	7.56±1.66	0.029*
	Lym×10^3^/μL	1.65±0.61	2.07±0.56	0.072
	NLR	7.37±3.22	3.78±1.09	0.007*
	Hbg g/dL	10.22±1.48	12.38±0.89	0.007*
Six hours after delivery				
	WBC×10^3^/μL	13.77±3.96	15.61±4.71	0.482
	Neu×10^3^/μL	11.23±3.81	12.05±5.70	0.820
	Lym×10^3^/μL	1.10±0.37	2.55±3.20	0.024*
	NLR	11.35±6.36	8.88±3.45	0.553
	Hbg g/dL	9.57±1.50	10.63±1.44	0.120

WBC: white blood cell; Neu: neutrophil; Lym: lymphocyte; NLR: neutrophil/lymphocyte ratio; Hbg: hemoglobin.

* p<0.05.

**Table 3:** Comparison of hemogram and demographic parameters according to the gestational weeks of delivery in group 3.

Group 3		Gestational weeks of delivery		P
		≤224 days (n=26)	>224 days (n=12)	
Mean±SD				
	Maternal age (years)	33.58±7.93	36.17±6.18	0.343
	Gravid	2.11±1.45	2.83±2.32	0.555
	Parity	0.53±0.84	1.17±2.40	0.999
	Hospitalization (days)	7.50±11.7	4.0±2.34	0.792
	Gestational days at cerclage applied	142.81±29.54	146.75±34.85	0.865
	Cerclage application-latency period (days)	25.69±21.75	114.25±55.53	<0.001*
Before cerclage application				
	WBC×10^3^/μL	12.19±2.73	9.84±2.52	0.005*
	Neu×10^3^/μL	9.59±2.32	7.31±2.02	0.008*
	Lym×10^3^/μL	1.85±0.63	1.81±0.59	0.963
	NLR	5.78±2.51	4.45±1.97	0.129
	Hbg g/dL	12.07±1.53	12.19±1.07	0.988
Six hours after cerclage application				
	WBC×10^3^/μL	13.02±3.83	9.58±2.40	0.028*
	Neu×10^3^/μL	10.58±3.95	7.69±2.60	0.039*
	Lym×10^3^/μL	1.79±0.61	1.49±0.70	0.250
	NLR	7.52±6.82	6.85±4.98	0.999
	Hbg g/dL	10.89±1.22	10.93±0.84	0.917
Before delivery				
	WBC×10^3^/μL	14.85±4.04	10.15±3.22	0.005*
	Neu×10^3^/μL	12.30±4.05	7.77±2.98	0.004*
	Lym×10^3^/μL	1.78±0.88	1.95±0.66	0.512
	NLR	9.01±6.01	4.29±1.68	0.034*
	Hbg g/dL	11.86±1.21	11.82±1.68	0.753
Six hours after delivery				
	WBC×10^3^/μL	16.53±5.44	16.45±6.82	0.677
	Neu×10^3^/μL	14.22±5.25	14.27±6.47	0.677
	Lym×10^3^/μL	1.47±0.44	1.53±0.91	0.853
	NLR	10.02±3.77	21.85±34.7	0.129
	Hbg g/dL	10.94±1.60	10.80±1.56	0.547

WBC: white blood cell; Neu: neutrophil; Lym: lymphocyte; NLR: neutrophil/lymphocyte ratio; Hbg: hemoglobin.

* p<0.05.

In Group 1, there were 12 (13%) pregnant women ≤32 gestational weeks, 9 (9.8%) pregnant women between >32 and ≤36 gestational weeks, and 71 (77.2%) pregnant women with gestational weeks >36 who have given birth. In Group 2, there were 11 (35.5%) pregnant women ≤32 gestational weeks, 5 (16.1%) pregnant women between >32 and ≤36 gestational weeks, and 15 (48.4%) pregnant women with gestational weeks >36 who have given birth. In Group 3, there were 26 (68.4%) pregnant women ≤32 gestational weeks, 6 (15.8%) pregnant women between >32 and ≤36 gestational weeks, and 6 (15.8%) pregnant women with gestational weeks >36 who have given birth.

In Group 1 (Table 1), early preterm delivery showed lower hemoglobin at the sixth hour after delivery (p=0.013). Other parameters, including WBC, neutrophil, lymphocyte, NLR, and Hbg values, were similar between groups at various time points.

Group 2 (Table 2) demonstrated higher NLR and lower hemoglobin concentrations before cerclage in deliveries below 32 weeks (p=0.002; p=0.018). Elevated NLR and WBC values persisted at the sixth hour after cerclage (p=0.048; p=0.042), and both NLR and neutrophil values before delivery were higher in early preterm delivery (p=0.007; p=0.029).

Group 3 (Table 3) indicated higher WBC and neutrophil levels before and after cerclage in early preterm delivery, while NLR levels remained similar. However, the NLR value before delivery was higher in individuals with early preterm delivery (p=0.034). Postpartum hemoglobin values did not significantly differ between groups.

Receiver operating characteristic analyses for Group 2 revealed an NLR cutoff of 4.75 before cerclage placement for predicting early preterm delivery (area=0.836, std. error=0.084, p=0.002, confidence interval; 95%). ROC analyses for Group 3, a WBC count equal to or exceeding 13.05×10^3^/μL before cerclage, predicted early preterm delivery (area=0.762, std. error=0.076, p=0.010, confidence interval; 95%).

## DISCUSSION

The key findings from our study suggest that hemogram parameters, when assessed based on obstetric history, are not predictive of early preterm delivery in pregnant women who have undergone prophylactic cerclage. Instead, these parameters have demonstrated predictive value for early preterm delivery in cases where emergency cerclage is performed due to cervical shortening and/or dilation.

Specifically, in situations where cervical length is <5 mm and/or cervical dilation is £3 cm, the NLR value emerges as a useful predictor for early preterm delivery. Conversely, in pregnant women undergoing emergency cerclage with cervical dilation exceeding 3 cm, the WBC value proves valuable in predicting early preterm delivery. These distinctions highlight the importance of tailoring predictive measures based on the specific circumstances surrounding cerclage procedures.

The study highlights the significance of elevated WBC values, a high NLR, and increased levels of interleukin-6 (IL-6) and interleukin-8 (IL-8) in amniotic fluid as indicators with predictive value for preterm delivery^6^. Additionally, cervical dilation, vaginal prolapse of the amniotic membrane, and cervical funneling are identified as key indicators for predicting preterm delivery subsequent to cerclage procedures^11,12^.

Our findings suggest that the predictive significance of various inflammatory markers for preterm delivery varies based on the degree of cervical dilation. Specifically, maternal blood WBC and NLR values determined before emergency cerclage can effectively predict early preterm delivery. However, as the degree of cervical dilation detected before cerclage increases, the WBC value becomes more meaningful in predicting preterm delivery.

Furthermore, in a scoring system utilized for prognostic assessment in emergency cervical cerclage procedures, higher scores are assigned with an increase in the amount of cervical dilation and a WBC value exceeding 13.60×10^3^/μL. This underscores the importance of considering both cervical dilation and WBC values in assessing the prognosis of emergency cerclage interventions^13^. In pregnant women who underwent cerclage due to cervical dilation exceeding 3 cm, the increase in WBC values before the cerclage procedure holds greater significance in predicting preterm delivery. The ROC analysis conducted for this patient group determined the WBC cutoff value for predicting early preterm delivery as 13.05×10^3^/μL.

In pregnant women who underwent cerclage due to cervical shortening (<5 mm) or cervical dilation level ≤3 cm, the increase in NLR values before the cerclage procedure holds greater significance in predicting preterm delivery. The ROC analysis conducted for this patient group determined the NLR cutoff value for predicting early preterm delivery as 4.75. Notably, a similar planned study demonstrated a cutoff value of 4.8 for this parameter^14^.

Second, the findings suggest that in Group 2 and Group 3, where emergency cerclage was applied and resulted in preterm delivery, the pre-delivery NLR value is statistically higher. In the group that underwent prophylactic cerclage, however, the pre-delivery NLR value did not exhibit statistically significant differences. It is conceivable that in cases of prophylactic cerclage, the underlying cause of preterm delivery may be non-intrauterine infection-related.

Another significant result is that in pregnant women who underwent emergency cerclage, the detection of a high NLR value during pregnancy follow-ups could serve as a predictor of preterm delivery. Additionally, the study indicates that the treatment of intrauterine infections leads to an improved obstetric prognosis when predicting preterm delivery. Furthermore, the use of antibiotic therapy in pregnancies, especially those with the presence of amniotic sludge, has been shown to improve the timing of childbirth^15^. The study concludes that the presence of amniotic sludge in patients with cervical shortening may function as an indicator for predicting preterm delivery^16^. In the monitoring of pregnant women undergoing emergency cerclage, the observation of a high NLR value can be utilized as an indicator for predicting preterm delivery. However, in the case of prophylactic cerclage applied based on obstetric history, neither pre-cerclage nor pre-delivery hematological parameters showed statistically significant data that could be used in predicting preterm delivery.

It has been demonstrated that in pregnant women who underwent prophylactic cerclage, second-trimester cervical length measurements and elevated plasma cytokine levels in maternal blood could potentially serve as data for predicting preterm delivery, contributing to forecasting the prognosis of pregnancy^17,18^. However, in our study, we have concluded that none of the hematological parameters we evaluated can be used for prediction.

In conclusion, our study suggests that the use of hemogram parameters for predicting early preterm delivery in pregnant women undergoing history-based prophylactic cerclage is not appropriate. However, for pregnant women who undergo cerclage due to cervical shortening or dilation, the NLR value can effectively predict early preterm delivery when cervical dilation is 3 cm or less and/or cervical shortening is 5 mm or less. Conversely, when cervical dilation exceeds 3 cm, it is more appropriate to use the WBC value to predict early preterm delivery.

## ETHICS COMMITTEE APPROVAL

The study was performed in accordance with the ethical standards for human research established by the Declaration of Helsinki and Good Clinical Practice guidelines and was approved by the local Ethics Committee of Süleyman Demirel University School of Medicine.

## INFORMED CONSENT

Informed consent was obtained from all participants, the purpose of the study was explained to them, and their participation was voluntary. Participants were also informed about their right to withdraw at any time without any negative consequences.
